# Association between the transcriptional levels of Htr-1a and tryptophan hydroxylase-1 in the hippocampus and the antifatigue effects of leucine on rats with postoperative fatigue

**DOI:** 10.3892/etm.2014.1973

**Published:** 2014-09-17

**Authors:** TIANTIAN WU, JING CHEN, JIANG ZHU, ZHEN YU

**Affiliations:** 1 Department of Gastrointestinal Surgery, The First Affiliated Hospital, Wenzhou Medical University, Wenzhou, Zhejiang 325035, P.R. China; 2 Department of Rheumatology, The First Affiliated Hospital, Wenzhou Medical University, Wenzhou, Zhejiang 325035, P.R. China; 3 Department of General Surgery, Shanghai Tenth People’s Hospital Affiliated to Tongji University, Shanghai 200072, P.R. China

**Keywords:** leucine, postoperative fatigue, open-field test, Htr-1a, tryptophan hydroxylase-1, quantitative polymerase chain reaction

## Abstract

Leucine (Leu), a branched-chain amino acid (BCAA), is widely used in clinical practice following severe burns, gastrointestinal surgery, trauma and sepsis. In the present study, the antifatigue effects of BCAAs on a postoperative fatigue (POF) rat model, induced by 70% intestinal resection, were investigated. Leu (16.5 g/l) was administered intraperitoneally at a dose of 18 ml/kg/day. The fatigue level and antifatigue effects of Leu were evaluated by open-field testing on day 1, 3, 5 and 7 after surgery. In addition, mRNA specimens were extracted and measured using a quantitative polymerase chain reaction method. The open-field test results indicated that Leu exhibited a significant antifatigue effect. The total distance travelled and the number of times the rats passed from the outermost grids of an open-top case were greatly improved in the Leu treatment group when compared with the POF model group. With the exception of the normal group, the mRNA expression levels of Htr-1a exhibited a similar trend in all other groups, reaching a climax on day 3 and 5, while being restored to a normal level on day 7. With regard to the Leu intervention group, the mRNA expression level of Htr-1a decreased significantly on day 3 and 5 following surgery. The mRNA expression levels of tryptophan hydroxylase-1 were unchanged in this short time period; however, the levels were increased gradually in the Leu treatment group. Therefore, Leu exhibited an apparent antifatigue effect on various 5-hydroxytryptamine-associated genes.

## Introduction

Currently, recovery in the postoperative period is receiving increasing attention. The concept of postoperative fatigue (POF) was first proposed by Professor Christensen (1), and is a common complication following surgery, particularly after enteral surgery. The fatigue manifests as central and peripheral symptoms. Depression, concentration difficulties, lethargy and weakness may be present for one to three months, even following uncomplicated abdominal surgery (2). POF increases treatment costs after surgery and burdens patients and their families. Etiological studies of POF have proposed that the condition is an endocrine-metabolic response to surgical and anesthetic stress. The behavioural and subjective changes during the postoperative convalescence are assumed to be the the physiological and metabolic consequences of surgery (3); therefore, developing a systemic therapy to abate POF and shorten the recovery period is desirable in surgery.

Leucine (Leu) is one of the three branched-chain amino acids (BCAAs; leucine, isoleucine and valine), which are essential amino acids. BCAAs, used as nutrient supplements, have an anticatabolic effect in the recovery period after exercise and decrease the muscle damage (4). BCAAs also contribute to the recovery from fatigue via the chemical reduction of tryptophan (Trp), allowing the molecule to pass through the blood-brain barrier and form the neurotransmitter, 5-hydroxytryptamine (5-HT) (5). Currently, BCAAs are used worldwide as nutrient supplements in bodybuilding and medicine, since further evidence has demonstrated that they accelerate the rate of myoprotein synthesis. Leu-only-supplemented nutrition was found to be promising, although further evaluation is required (6). However, the Trp-5-HT-central fatigue hypothesis, which is based on the principles of depression, anhypnia and dementia, has not been fully acknowledged (7).

5-HT was found to be a relative frequent fatigue factor in 1987, with its synthesis and reactive receptors contributing to the feeling of fatigue (central fatigue) (8). Observations from studies on sports fatigue revealed that the presence of high concentrations of 5-HT in the hippocampi of rats led to fewer symptoms of depression, such as malaise, somnolent, lethargy and debilitation (9–11). This may be caused by factors associated with synthesis. In a previous study, the total concentration of 5-HT in the brains of different postoperative rats were identified to have a similar trend (12). Therefore, the aim of the present study was to identify the expression levels of the tryptophan hydroxylase-1 (TPH-1) gene, the crucial enzyme in the pathway of 5-HT synthesis in the brain, and the 5-HT receptor 1A (Htr-1a) gene, the main receptor of 5-HT in the brain. In addition, the study analyzed the association between POF and these gene expression levels.

## Materials and methods

### Animals

In total, 128 adult specific-pathogen free male Sprague-Dawley rats (weight, 430±20 g) were obtained from Beijing Vital River Laboratories (Beijing, China). Experimental procedures were approved by the Institutional Animal Committee of Wenzhou Medical University (Wenzhou, China). All the rats in the experiment were treated according to the ‘Guide for the Care and Use of Laboratory Animals’, and were maintained in specific-pathogen free conditions throughout the experiment. The temperature was controlled at 20–25°C, the humidity was maintained at 45–55% and the lighting was 10 h light/14 h dark per cycle. The rats were fed normally, with the exception of the days prior to and following surgery when they were forbidden to eat.

### Grouping and surgery

**Figure 1:**
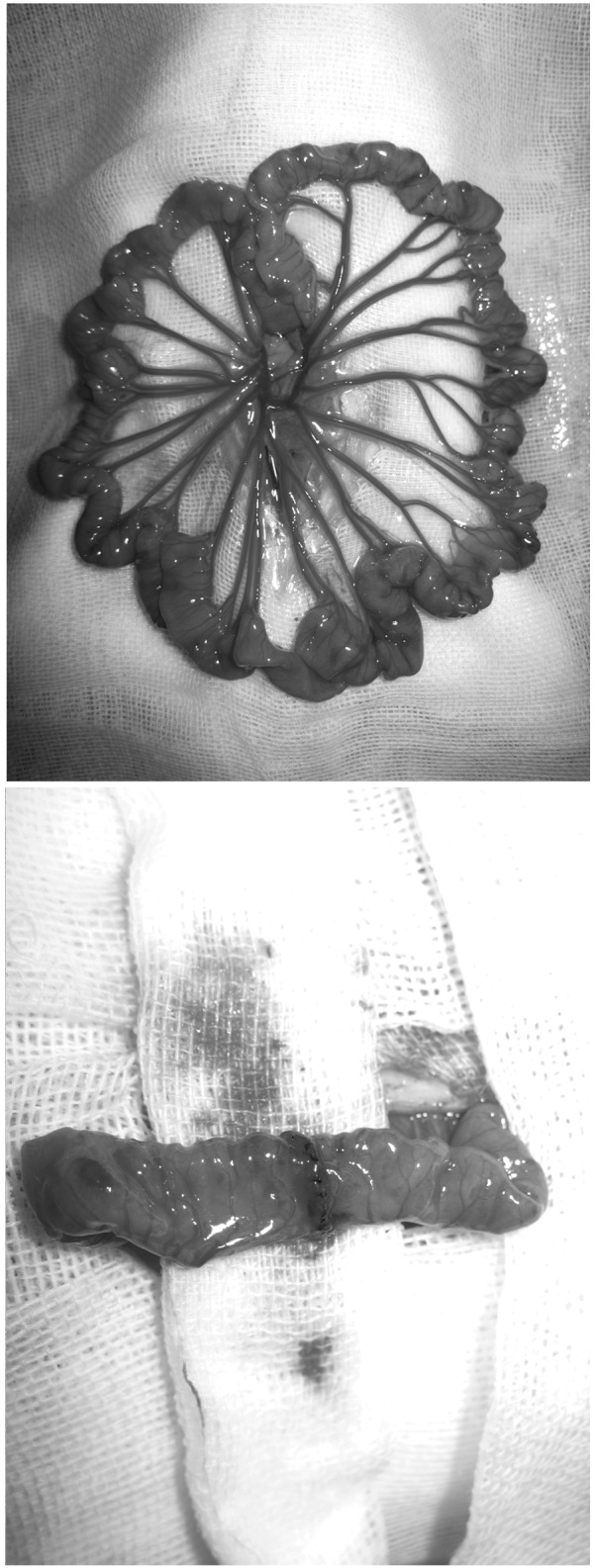
Mesenteric vessels were ligated, 70% of the intestine was cut and the remnant was anastomosed.

The rats were divided randomly into groups of 32 rats each: Normal group (NG; no interventions); sham operation group (SG; opened and closed abdomen); POF model group (MG; 70% small intestinal resection) (11) and Leu-treated POF model group (LG; treated with Leu on the basis of the MG). Each group was subdivided into day 1, 3, 5 and 7 subgroups, which indicated the number of days after surgery (for the NG, there was no change among the subgroups as no surgery was carried out). All the rats were anesthetized by an abdominal injection of 10% chloral hydrate (0.35 ml/kg) prior to surgery. Rats in the LG group were administered Leu (Sigma-Aldrich, St. Louis, MO, USA) dissolved in saline solution (16.5 g/l), at a dose of 18 ml/kg/day, by abdominal injection. All rats were fed normally prior to and following surgery (with the exception of the fasting days the days before and after surgery). The 70% small intestinal resection is illustrated in Fig. 1.

### Open-field test

Each rat was placed in the center of one of the 25 equal squares of an open-top black case (100 × 100 × 50 cm). The motion curve, total distance travelled and the number of times the rat passed through the outermost grids and central square in 5 min were recorded using an image acquisition system (Sony HDR-CX220E; Sony Corporation, Tokyo, Japan). Counting these frequencies and the number of times that the rats cleaned their face, mouth and head with their forelimb was performed manually.

### Quantitative polymerase chain reaction (qPCR)

Hippocampus specimens were obtained after perfusion from the left ventricle using normal saline solution. Total RNA was extracted from the hippocampus tissues with TRIzol reagent (Invitrogen Life Technologies, Grand Island, NY, USA). The extracted RNA samples were precipitated in isopropanol and incubated at −20°C overnight. Next, the samples were dissolved in diethylpyrocarbonate-treated water and stored at −80°C. An ultraviolet spectrophotometer (DU-800; Beckman Coulter^®^, Miami, FL, USA) was used for quantification and qualification. The RNA samples used in this experiment had an A260/A280 ratio in the range of 1.8–2.0. The integrity of the RNA was identified using agarose gel electrophoresis (13). Next, 1 μl RNA (1,000 ng/μl) was reverse transcribed to 20 μl cDNA using a reverse transcription kit (Toyobo Corporation, Osaka, Japan). Glyceraldehyde-3-phosphate dehydrogenase (GAPDH), a housekeeping gene, was selected as the internal control. According to the manufacturer’s instructions provided in the SYBR^®^ Green kit (Toyobo Corporation), the qPCR reaction mixtures (10 μl) of the genes, GAPDH, TPH-1 and Htr-1a, consisted of 1 μl cDNA, 0.6 μl sense primer (10 μM; Invitrogen Life Technologies), 0.6 μl antisense primer (10 μM; Invitrogen Life Technologies), 5 μl SYBR^®^ Green (Toyobo Corporation), 1 μl buffer and 1.8 μl ddH_2_O. Thermal cycling was performed in a 10-μl volume with a 30-sec preheating step at 95°C, followed by 40 cycles of 5 sec at 95°C, 10 sec at 55°C and 15 sec at 72°C, following which a melting curve was constructed. The sense and antisense sequences of each gene are shown in Table I.

### Statistical analysis

Statistical analysis was performed using SPSS 20.0 software (IBM, Armonk, NY, USA) for Windows. Open-field test results are expressed as the mean ± standard deviation, and statistical significance was determined by one-way analysis of variance and the least significant difference t-test. The results from the qPCR are expressed as 2^−ΔΔCt^, and were analyzed with the Student’s t-test. P<0.05 was considered to indicate a statistically significant difference.

## Results

### Results of open-field testing

The behavior of the rats in the MG was apathetic compared with the rats in the NG, showing decreased activity, hair loss and poor hygiene due to the lack of cleaning themselves. However, rats in the SG were more active compared with the MG rats on day 3, 5 and 7 of the postoperative period. The injection of Leu improved the behavior of the rats in the LG when compared with the MG rats. The results of the open-field test are shown in Table II.

The total distance travelled and the number of times passing from the outermost grids for the SG rats were significantly lower compared with the NG rats on day 1 and 3 after surgery. However, the exploration periods of the SG rats were only significantly lower than that of the NG rats at day 1 after surgery. MG rats exhibited a significant reduction in the total distance travelled when compared with the NG rats on day 1, 3 and 5. In addition, the number of times the MG rats passed through the outermost grids was significantly lower compared with the NG rats on day 1 and 3. The exploration periods of the MG rats showed no differences, with the exception of day 1 after surgery. The total distance travelled and the number of times the outermost grids were passed by the LG and MG rats on days 1 and 3 were similar. Certain indices returned to their normal levels in the LG rats at day 5 after surgery. The total distance travelled and the number of times the outermost grids were passed by the LG rats improved significantly on day 5 and 7, when compared with the MG rats.

### Results of qPCR

Relative quantification was employed to perform qPCR, and the results are expressed in terms of 2^−ΔΔCt^ (Table III). Data for the Htr-1a gene indicated that the transcriptional levels of all the groups on day 1 and 7 after surgery were not significantly different from the NG. With the exception of the NG group, all groups followed a similar trend, showing an increase on day 3, and a decrease on day 5. Statistically significant differences were not observed between the SG and MG on these two days; however, the LG exhibited statistically significant decreases when compared with these groups at these time points. With regard to the TPH-1 gene, the transcriptional levels in the SG and MG were similar for the whole postoperative period. By contrast, the transcriptional level in the LG increased significantly on day 5 and 7 after surgery; however, the difference between these two days was not significant.

## Discussion

POF is a common postoperative symptom, appearing particularly after uncomplicated abdominal or thoracic surgery. Research on POF has helped shorten the recovery period and improve the quality of life following surgery. In a previous study, a fatigue model induced by 70% small intestinal resection was proposed (14). The present study demonstrated that the intervention of Leu upon this model induced an antifatigue effect. In addition, open-field testing revealed that Leu enhanced the activity of rats after surgery.

Previous studies have identified that the peripheral effects of 5-HT on neural networks inhibit sensory-generating and sensory-integrating processes, facilitate motor output and suppress the somatosensory system (15–18). However, the influence of 5-HT in the brain is more complicated. 5-HT deficiency has been hypothesized to be a cause of depression, which is supported by pharmacological studies in conjunction with etiological findings (19–21). By contrast, the levels of 5-HT in the anterior hypothalamic and hippocampal areas are increased in rats with fatigue caused by excessive exercise, surgery or diseases, such as cholestasis and neoplasm (22–24). When the concentration of 5-HT in the aforementioned tissues is decreased using pharmacological methods, the physical state is improved (11).

The present study investigated whether the transcriptional level of 5-HT-related genes changes in the short period following surgery in rats. Regardless of the administration of Leu, the mRNA expression levels of Htr-1a changed in the postoperative period, increasing significantly on day 3 and 5 after surgery, and returning to normal levels on day 7. When Leu was administered, the expression levels decreased significantly on day 3 and 5, as compared with the MG. However, the mRNA expression levels of TPH-1 were not altered unless Leu was injected into the abdominal cavity. The Htr-1a gene encodes mRNA that transcribes a G-protein coupled receptor on the pre- and postsynaptic membrane, which regulates the biosynthesis of downstream inhibitory substances (25). The TPH-1 gene encodes mRNA that transcribes an enzyme that catalyzes 5-HT synthesis and is the most predominant synthetase of 5-HT. Changes in the mRNA expression of Htr-1a were negatively correlated with the results of the open-field test. These gene activities indicate that fatigue after enteral surgery depends on changes in the expression of Htr-1a within a short postoperative period. However, when Leu was administered daily for a relatively long period, the gene expression of TPH-1 changed accordingly on day 5 and 7. Therefore, surgery may not alter the transcriptional level of the TPH-1 gene.

5-HT receptors modulate the release of numerous neurotransmitters (with the exception of serotonin), including glutamate, GABA, dopamine and epinephrine, as well as a number of hormones, including oxytocin, prolactin, vasopressin and cortisol (26). 5-HT 1A receptors are the most common 5-HT receptors. In areas such as the hippocampus, amygdala and septum, 5-HT 1A receptors function as postsynaptic receptors (27). The present study demonstrated that therapy with Leu may decrease the density of 5-HT 1A receptors on postsynaptic membranes; thus, abate the inhibitory affect of 5-HT in neuromodulation. Additionally, Leu therapy may increase TPH-1 density in neurons, and thus, control the synthesis of 5-HT.

In conclusion, Leu has been found to have an antifatigue effect on a rat model of POF induced by 70% intestinal resection. Although numerous pharmacological mechanisms exist, the present study investigated the transcription of two genes in the seven-day period after surgery. However, further research is required since neurotransmitters, other associated genes and the expression of these genes remain unknown.

## Figures and Tables

**Table I:** Sequences of the Htr-1a and TPH-1 genes.

Gene name	Sense sequence (5′-3′)	Antisense sequence (5′-3′)
Htr-1a	gtgccggcctgctttcttcc	gcgctggttatgctcttgctgtct
TPH-1	atggggcagtgaggcagggtgacg	ccaagcaggcgggggcataggagt
GAPDH	ggtgctgagtatgtcgtgga	gccatgccagtgagcttccc

TPH-1, tryptophan hydroxylase-1; GAPDH, glyceraldehyde-3-phosphate dehydrogenase.

**Table II:** Results of the open-field test.

		Horizontal movement			Vertical movement	
			
Groups	Rats (n)	Total distance travelled (m)	Times passing from the outermost grids (n)	Times passing from central square (n)	Explorations (n)	Clean ups (n)
NG-D1	8	19.00±3.79	104.4±42.2	2.0±0.8	12.6±7.4	2.8±2.1
NG-D3	8	17.24±4.76	112.4±38.9	2.1±1.1	14.3±6.2	2.6±1.2
NG-D5	8	18.36±5.21	96.59±26.0	1.8±1.0	16.8±4.7	1.8±1.6
NG-D7	8	21.6±3.86	117.5±19.8	2.0±1.2	12.6±6.5	2.6±1.4
SG-D1	8	11.51±2.66^a^	71.1±13.9^a^	2.1±1.1	6.4±3.9^a^	1.9±1.7
SG-D3	8	15.33±2.85^a^	104.9±21.8^a^	1.6±0.9	12.4±5.0	2.6±2.4
SG-D5	8	19.12±2.40	139.0±31.7	2.0±1.1	13.0±5.8	3.6±2.8
SG-D7	8	18.42±3.93	121.5±24.8	1.8±1.0	17.3±6.2	3.5±2.2
MG-D1	8	10.52±4.98^a^	60.0±18.9^a^	1.1±0.4	4.6±2.6^a^	2.6±2.4
MG-D3	8	9.75±2.59^a^	69.3±19.0^a^	0.5±0.5	9.0±4.0	2.1±1.7
MG-D5	8	12.69±2.07^a^	118.0±15.7	1.0±0.0	13.4±5.4	3.3±1.7
MG-D7	8	17.34±3.50	139.3±23.9	1.3±0.5	15.9±6.9	3.3±2.4
LG-D1	8	9.06±2.06^a^	61.4±16.1^a^	0.9±0.4	5.9±2.2^a^	3.9±2.5
LG-D3	8	11.11±3.06^a^	77.9±24.5^a^,^b^	0.8±0.5	9.9±3.3	2.9±1.4
LG-D5	8	19.82±2.42^b^	136.4±19.1^b^	0.9±0.6	15.1±4.9	5.1±3.4
LG-D7	8	23.21±3.63^b^	153.3±23.4^b^	1.0±0.5	19.6±6.9	4.9±3.5

Results are expressed as mean ± standard deviation.

a P<0.05, vs. NG-D1

b P<0.05, vs. MG at the same parameter.

NG, normal group; SG, sham operation group; MG, POF model group; LG, Leu-treated POF model group; Dn, day n (where n=1,3,5 or 7); Leu, leucine.

**Table III:** Results of qPCR.

Groups	Rats (n)	Htr-1a	TPH-1
MG-D1	8	1.0364±0.3998^a^	1.4710±0.6517
MG-D3	8	40.1413±14.5137^b^	0.8461±0.6696
MG-D5	8	21.8495±7.8794^b^	1.4303±0.9012
MG-D7	8	1.5624±1.5698^a^	3.1004±1.4620
SG-D1	8	1.5274±1.3713^a^	2.9534±1.2771
SG-D3	8	47.2402±19.7972^b^	0.6509±0.2823
SG-D5	8	25.6668±8.6453^b^	1.3636±0.5345
SG-D7	8	1.2369±0.9885^a^	0.8305±0.4100
LG-D1	8	1.4380±1.1323^a^	1.2571±0.9843
LG-D3	8	25.3650±6.3114^c^	5.6938±1.0210
LG-D5	8	11.2531±3.7433^c^	10.2159±1.2561
LG-D7	8	1.2406±1.2125^a^	11.1382±1.3120

Relative expression levels were calculated using the 2^−ΔΔCt^ method and are expressed as the mean ± standard deviation.

a P>0.05, between day 1 and 7 after surgery

b P>0.05, between day 3 and 5 after surgery

c P<0.05, vs. MG values. For the TPH-1 gene, the values of LG-D5 and LG-D7 were higher compared with the other subgroups (P<0.05); however, there was no significant difference between the values at D5 and D7 (P>0.05).

SG, sham operation group; MG, POF model group; LG, Leu-treated POF model group; Dn, day n (where n=1,3,5 or 7); qPCR, quantitative polymerase chain reaction; TPH-1, tryptophan hydroxylase-1; Leu, leucine.

## References

[1] Christensen T, Kehlet H (1993). Postoperative fatigue. World J Surg.

[2] Rubin GJ, Hardy R, Hotopf M (2004). A systematic review and meta-analysis of the incidence and severity of postoperative fatigue. J Psychosom Res.

[3] Salmon P, Hall GM (1997). A theory of postoperative fatigue: an interaction of biological, psychological, and social processes. Pharmacol Biochem Behav.

[4] Gastmann U, Lehmann MJ (1998). Overtraining and the BCAA hypothesis. Med Sci Sports Exerc.

[5] Newsholme EA, Blomstrand E (2006). Branched-chain amino acids and central fatigue. J Nutr.

[6] De Bandt JP, Cynober L (2006). Therapeutic use of branched-chain amino acids in burn, trauma, and sepsis. J Nutr.

[7] Åstrand PO, Rodahi K, Dahl H, Strømme SB (2003). Textbook of Work Physiology: Physiological Bases of Exercise.

[8] Newsholme EA, Acworth IN, Blomstrand E, Benzi G (1987). Amino acids, brain neurotransmitters and a functional link between muscle and brain that is important in sustained exercise. Advances in Myochemistry.

[9] López-Muñoz F, Alamo C (2009). Monoaminergic neurotransmission: the history of the discovery of antidepressants from 1950s until today. Curr Pharm Des.

[10] Jacobsen JP, Medvedev IO, Caron MG (2012). The 5-HT deficiency theory of depression: perspectives from a naturalistic 5-HT deficiency model, the tryptophan hydroxylase 2Arg439His knockin mouse. Philos Trans R Soc Lond B Biol Sci.

[11] Tan S, Zhou F, Li N (2013). Anti-fatigue effect of ginsenoside Rb1 on postoperative fatigue syndrome induced by major small intestinal resection in rat. Biol Pharm Bull.

[12] Dong QT, Zhou F, Yu Z, Tan SJ, Wang Q, Zhang XD (2011). Association of the changes of central serotonin and peripheral blood free amino acids with postoperative fatigue after abdominal surgery. Zhonghua Wei Chang Wai Ke Za Zhi.

[13] Feng HZ, Wei B, Jin JP (2009). Deletion of a genomic segment containing the cardiac troponin I gene knocks down expression of the slow troponin T gene and impairs fatigue tolerance of diaphragm muscle. J Biol Chem.

[14] Zhang XD, Chen BC, Dong QT (2011). Establishment and assessments of a new model for the postoperative fatigue syndrome by major small intestinal resection in rats. Scand J Gastroenterol.

[15] Barbeau H, Rossignol S (1990). The effects of serotonergic drugs on the locomotor pattern and on cutaneous reflexes of the adult chronic spinal cat. Brain Res.

[16] Christenson J, Franck J, Grillner S (1989). Increase in endogenous 5-hydroxytryptamine levels modulates the central network underlying locomotion in the lamprey spinal cord. Neurosci Lett.

[17] Harris-Warrick RM, Cohen AH (1985). Serotonin modulates the central pattern generator for locomotion in the isolated lamprey spinal cord. J Exp Biol.

[18] Bizzi E, Giszter SF, Loeb E, Mussa-Ivaldi FA, Saltiel P (1995). Modular organization of motor behavior in the frog’s spinal cord. Trends Neurosci.

[19] Lapin IP, Oxenkrug GF (1969). Intensification of the central serotoninergic processes as a possible determinant of the thymoleptic effect. Lancet.

[20] Coppen A (1967). The biochemistry of affective disorders. Br J Psychiatry.

[21] Mendels J, Stinnett JL, Burns D, Frazer A (1975). Amine precursors and depression. Arch Gen Psychiatry.

[22] Burak KW, Le T, Swain MG (2001). Increased midbrain 5-HT1A receptor number and responsiveness in cholestatic rats. Brain Res.

[23] Terzioğlu B, Aypak C, Yananlı HR (2006). 5-hydroxytryptamine release in the anterior hypothalamic and the hippocampal areas of cholestatic rats. Life Sci.

[24] Uusitalo AL, Valkonen-Korhonen M, Helenius P (2004). Abnormal serotonin reuptake in an overtrained, insomnic and depressed team athlete. Int J Sports Med.

[25] Sullivan GM, Ogden RT, Huang YY (2013). Higher in vivo serotonin-1A binding in posttraumatic stress disorder: a PET study with [11C]WAY-100635. Depress Anxiety.

[26] Ito H, Halldin C, Farde L (1999). Localization of 5-HT1A receptors in the living human brain using [carbonyl-11C]WAY-100635: PET with anatomic standardization technique. J Nucl Med.

[27] Peroutka SJ (1988). 5-Hydroxytryptamine receptor subtypes. Annu Rev Neurosci.

